# Promoting critical thinking in undergraduate sensation and perception

**DOI:** 10.3389/fpsyg.2025.1712565

**Published:** 2025-12-11

**Authors:** Nicole D. Anderson

**Affiliations:** Department of Psychology, MacEwan University, Edmonton, AB, Canada

**Keywords:** critical thinking, sensation and perception, psychology education, pseudoscience, teaching demonstrations

Critical thinking is an essential skill for students to develop given the modern environment of misinformation and disinformation. The ability to evaluate information validity is highly valued by both academic institutions and employers (e.g., Haber, 2020), and over the past decade there has been a growing movement to incorporate critical thinking across disciplines. Research consistently shows that integrating evidence-based thinking and problem-solving into the classroom improves student understanding and engagement (e.g., Kim et al., 2013; Cargas et al., 2017; Tiruneh et al., 2018). Here, I suggest a collection of real-world products related to sensory processing that instructors can use to design classroom activities that promote critical thinking in sensation and perception.

Psychology is well-positioned to foster critical thinking, due to its emphasis on scientific reasoning and evidence evaluation. Psychology students must learn to analyze research methods and assess the validity of experimental designs, making critical thinking essential to their training. The discipline also offers opportunities to confront misinformation, particularly in the form of “pop psychology.” The book *50 Great Myths of Popular Psychology* (Lilienfeld et al., 2010) provides a compelling framework, presenting widely accepted but flawed beliefs that can be deconstructed through critical analysis. Using familiar examples that students may already believe or recognize captures their interest and provides an engaging entry point into evidence-based reasoning. Thus, psychology serves as an ideal vehicle for introducing and reinforcing critical thinking at the undergraduate level through active engagement.

One area of psychology that strongly benefits from active engagement is sensation and perception. This course can feel intimidating for many psychology students because of its interdisciplinary nature (Aivar, 2024), but much of that concern can be mitigated using instructor-led demonstrations and student-centered in-class activities. Demonstrations provide direct, observable illustrations of perceptual phenomena, while activities invite students to apply those principles through guided exploration and analysis. Both approaches help clarify abstract concepts and make the material more concrete. The value of demonstrations for sensation and perception is underscored by a recent special issue of *Visual Cognition* devoted to highlighting demonstrations that instructors can use (see Kosovicheva et al., 2024, for an overview). As emphasized throughout this issue, shared collections of teaching resources that include demonstrations accompanied by readings, discussion prompts, and creative projects improve teaching effectiveness and promote deep thinking. Importantly, both demonstrations and activities serve as powerful vehicles for fostering critical thinking.

Consumer products that claim to influence perception serve as effective tools for in-class critical thinking activities in sensation and perception. Their natural appeal to students and the general public is evident in the popularity of products marketed to enhance or restore sensory abilities, whether for vision, pain, or other perceptual conditions. Many of these products lack scientific support, and analyzing their claims in relation to established sensory mechanisms deepens students' understanding. Conversely, products that appear implausible yet prove effective are especially engaging, as they prompt students to reason through why they work. Such contrasts between pseudoscientific and evidence-based tools both reinforce core concepts and foster critical thinking.

Here, I present a selection of real-world products, some grounded in scientific evidence and others not, that claim to influence various aspects of sensory functioning. These examples are intended as flexible resources for instructors who wish to design critical thinking activities. Legitimate products can be used to illustrate and reinforce key principles of sensory processing by exploring the scientific rationale behind their effectiveness, whereas pseudoscientific items offer opportunities for students to apply their knowledge to identify why such claims are unsupported. In my classes, students work in groups to evaluate a list of products, researching whether each claim aligns with what we have learned about that sensory system and then discussing their conclusions as a class. These same products could also be adapted for a “Psychbusters”-style assignment (Blessing, 2023), in which students investigate the accuracy of perceptual claims as a term project, or for a refutational infographic activity where students design an evidence-based infographic to debunk the product's claims (Laurin and Kelly, 2025). Even a brief analysis of these products can prompt meaningful reflection on the principles of sensory processing while encouraging students to develop evidence-based reasoning skills and the kind of critical analysis that is essential in both scientific and everyday contexts.

## Products for critical thinking in sensation and perception

In this section, I provide descriptions of two evidence-based and two pseudoscientific products. Each example includes a discussion of how it can be used as a teaching tool in sensation and perception. A broader list of additional products, with brief notes on their scientific viability, is provided in Table 1. It is important to emphasize that the effectiveness of some products may be condition-specific; evidence of benefit in one context does not necessarily generalize to others.

**Table 1:** Examples of products for critical discussion in sensation and perception.

**Sensory domain**	**Scientific support**	**Product**	**Claim**	**Critical thinking opportunity**
Vision	Evidence-based	Dark adaptation goggles	Adapt to low light conditions in a sighted environment	Discuss rod/cone spectral sensitivity and dark adaptation
		Orthokeratology	Temporarily reshapes cornea to correct myopia	Explore optical correction methods and the role of the cornea in eye optics
		Color-blind glasses	Enhance color discrimination for some forms of color blindness	Discuss different forms of color blindness and the effectiveness of wavelength filtering
	Pseudoscientific	Bates method	Eye exercises can cure vision problems without glasses	Explore causes of refractive error and dangers of UV overexposure
		Pinhole glasses	Permanently improve vision by forcing focus through pinholes	Contrast temporary optical effects with actual optical correction
Audition	Evidence-based	Bone conduction headphones	Transmit sound through bones of the skull	Explore the mechanical propagation of sound and alternative auditory pathways
		Cognitive behavioral therapy for tinnitus	Reduces tinnitus awareness and distress	Discuss the role of cognitive processes in sound perception
	Pseudoscientific	Ear candling	Draw out earwax and toxins using heated candles to improve hearing	Discuss differences in conductive and sensorineural hearing loss and risk/benefit analysis
		Chininum Sulphuricum	Homeopathic remedy for tinnitus relief	Discuss the placebo effect and perceptual interpretation in tinnitus
Somatosensation	Evidence-based	Vibration therapy	Transdermal vibrations inhibit pain	Discuss the role of lateral inhibition in how touch reduces pain perception (Gate control theory)
		Capsaicin cream	Capsaicin reduces nociceptor responsiveness	Explore the relationship between nociceptor pain and analgesia
	Pseudoscientific	Magnetic bracelets	Magnetic fields can relieve pain and improve circulation	Discuss the difference between mechanical and magnetic energy and somatosensation
		Copper compression gloves	Copper reduces arthritis pain through skin contact	Explore the role of chemicals in nociception
Olfaction/Gustation	Evidence-based	Olfactory training kits	Restore or improve sense of smell through repeated exposure	Discuss neuroplasticity and sensory rehabilitation
		Miracle fruit tablets	Alter taste perception by making sour foods taste sweet	Explore taste receptor physiology and modulation
	Pseudoscientific	Aromatherapy for anosmia	Essential oils restore smell after loss	Distinguish between olfactory training and unsupported scent-based claims
		Argentum Nitricum	Homeopathic treatment to reduce sugar cravings	Contrast placebo effects with claims of sensory adaptation

### Dark adaptation goggles

Dark adaptation goggles are red-tinted glasses marketed to promote rapid dark adaptation. These goggles allow primarily long-wavelength light to pass through, creating a red-filtered visual environment. The claim is that wearing the goggles under normal lighting conditions enables users to enter a dark environment fully adapted, bypassing the typical 25-to-30-min adjustment period required for dark adaptation. This can be advantageous in contexts that require a quick transition from light to dark, such as in military operations, hunting, or astronomy.

These goggles are an example of a legitimate sensory product grounded in well-established principles of vision. A foundational concept in sensation and perception is the dark adaptation curve, a non-linear function describing how light sensitivity changes over time in darkness. The early phase of the curve reflects cone adaptation, which adapt quickly but are not very light-sensitive. The later phase reflects rod adaptation. Rods take longer to adapt but ultimately become far more sensitive to low light. Full dark adaptation is reached once rod adaptation is complete.

Because rods are insensitive to long-wavelength light, wearing red-filtered goggles minimizes their stimulation, allowing them to regenerate even under otherwise lit conditions. Meanwhile, cones remain active, allowing users to function visually in red light without disrupting rod adaptation.

Using dark adaptation goggles as a teaching example enables students to critically examine the physiology of photoreceptor regeneration, the functional distinctions between rods and cones, and the spectral sensitivity of the visual system. It also encourages students to evaluate real-world applications of sensory science and the evidence behind marketed perceptual products.

### Pinhole glasses

Pinhole glasses are marketed as a non-prescription alternative to standard optical lenses. They consist of opaque black surfaces perforated with a grid of small holes that restrict incoming light. Manufacturers claim that wearing these glasses for extended periods will train the eyes to see more clearly over time. Some even suggest that consistent use will eliminate the need for prescription lenses altogether.

Although the concept of pinhole optics is based on valid optical principles, these glasses lack scientific support. In clinical settings, pinhole lenses are sometimes used to temporarily improve acuity by minimizing the effects of refractive error during diagnostic evaluations. However, this effect is purely optical and temporary. Pinhole glasses do not cause structural changes in the eye and cannot correct or improve vision once they are removed. Furthermore, because they block a large portion of the visual field, they significantly limit peripheral vision and light intake, making them impractical for daily use.

Critically examining the claims behind pinhole glasses provides students with an opportunity to explore how optical correction works, and why structural problems like refractive errors require structural solutions. This discussion reinforces key concepts in physiological optics, including how the cornea and lens shape incoming light and how visual acuity is affected by focal length errors. It also prompts reflection on how easily real scientific principles can be misapplied or misrepresented in the marketing of sensory products.

### Vibration therapy

Vibration therapy (also known as transcutaneous electrical nerve stimulation or TENS) applies mechanical oscillations to the skin to reduce pain. It is used for chronic pain, arthritis, neuropathic pain, and short-term relief in acute injuries. While many commercial products are marketed for home use, the technique is also applied in clinical settings.

Although vibration therapy may seem questionable because vibrations do not directly target nociceptors, it is a legitimate treatment supported by perceptual science. The key insight is that pain perception can be modulated without directly suppressing pain pathways.

Several theoretical explanations for its effectiveness have been proposed, all of which are grounded in concepts commonly taught in sensation and perception. One potential mechanism is the gate control theory of pain, which proposes that non-nociceptive input (i.e., tactile stimulation) can inhibit pain signals at the spinal cord. In this context, mechanical vibrations activate mechanoreceptors that may inhibit nociceptive transmission through lateral inhibition. Another possibility is that vibrations serve as a distraction, reducing attentional focus on the painful stimulus. This aligns with research on the modulatory role of cognitive processes in pain perception. For a deeper review of the mechanisms behind vibratory analgesia, see Hollins et al. (2014).

Discussing vibration therapy in the classroom provides an opportunity for students to explore how pain perception can be shaped by competing sensory input, spinal processing, and cortical modulation. It also encourages them to critically examine how real perceptual principles can explain the effectiveness of treatments that may initially seem implausible.

### Magnetic bracelets

Magnetic bracelets are wearable accessories marketed to relieve pain and inflammation through magnetic fields. A common claim is that the magnets influence the alignment or behavior of iron in the blood, thereby improving circulation and ultimately reducing pain. Magnetic elements are also embedded in other products such as socks, belts, and athletic wear, but bracelets remain among the most widely marketed and accessible forms.

Despite these claims, there is no scientific evidence supporting the use of magnetic bracelets for pain relief. Neurons in the human body are not responsive to magnetic energy in a way that would modulate pain. Even if magnets did influence blood flow, circulation is not the primary mechanism underlying pain perception, and the iron in blood is not magnetic. Furthermore, the magnetic fields used in consumer products are too weak to meaningfully affect biological processes. Therefore, the central claims of magnetic therapy products lack both biological plausibility and empirical support.

Discussing the viability of magnetic bracelets encourages students to critically examine the neural basis of pain and the kinds of environmental stimuli that can be transduced by the sensory systems. It also highlights the importance of distinguishing between biologically meaningful mechanisms and marketing claims.

## Conclusion

In this paper, I have presented examples of products that can be used to promote critical thinking in a sensation and perception course. A broader selection of products, with brief descriptions of their scientific viability, is provided in Table 1. Although this discussion has focused on products claiming to affect vision and pain perception, many others relate to the remaining senses. In the chemical senses, one particularly interesting example is the “miracle fruit” pill, available online, which offers a vivid demonstration of taste modulation. This example allows for discussion of the chemotransductive nature of gustation and how chemical properties of food can directly alter sensory experience. Another valuable category of products to examine is homeopathic treatments for sensory issues (e.g., *Chininum Sulphuricum* and *Argentum Nitricum*), which are particularly instructive given both the pseudoscientific foundations of homeopathy and the extraordinary claims these products make when considered in light of sensory processes.

Because students are often exposed to such products through social media, advertising, and online sources that lack peer review, they tend to find it especially engaging to evaluate these claims through a scientific lens. Critically examining these products not only helps reinforce key course concepts but also equips students with valuable real-world skills. In particular, it helps them learn how to assess health-related claims and become more informed consumers. Whether a product is evidence-based or pseudoscientific, using it as a teaching tool encourages deeper conceptual understanding and supports the development of scientific reasoning.
